# Astrocyte‐T cell crosstalk regulates region‐specific neuroinflammation

**DOI:** 10.1002/glia.23783

**Published:** 2020-01-21

**Authors:** Jessica L. Williams, Sindhu Manivasagam, Brandon C. Smith, Julia Sim, Lauren L. Vollmer, Brian P. Daniels, John H. Russell, Robyn S. Klein

**Affiliations:** ^1^ Department of Neurosciences, Lerner Research Institute Cleveland Clinic Foundation Cleveland Ohio; ^2^ Department of Medicine Washington University School of Medicine St. Louis Missouri; ^3^ Department of Developmental Biology Washington University School of Medicine St. Louis Missouri; ^4^ Department of Pathology and Immunology Washington University School of Medicine St. Louis Missouri; ^5^ Department of Anatomy and Neurobiology Washington University School of Medicine St. Louis Missouri

**Keywords:** astrocyte, CXCR7, cytokine, neuroinflammation, regional heterogeneity, T cell, VCAM‐1

## Abstract

During multiple sclerosis (MS), an inflammatory and neurodegenerative disease of the central nervous system (CNS), symptoms, and outcomes are determined by the location of inflammatory lesions. While we and others have shown that T cell cytokines differentially regulate leukocyte entry into perivascular spaces and regional parenchymal localization in murine models of MS, the molecular mechanisms of this latter process are poorly understood. Here, we demonstrate that astrocytes exhibit region‐specific responses to T cell cytokines that promote hindbrain versus spinal cord neuroinflammation. Analysis of cytokine receptor expression in human astrocytes showed region‐specific responsiveness to Th1 and Th17 inflammatory cytokines. Consistent with this, human and murine astrocytes treated with these cytokines exhibit differential expression of the T cell localizing molecules VCAM‐1 and CXCR7 that is both cytokine and CNS region‐specific. Using in vivo models of spinal cord versus brain stem trafficking of myelin‐specific T cells and astrocyte‐specific deletion strategies, we confirmed that Th1 and Th17 cytokines differentially regulate astrocyte expression of VCAM‐1 and CXCR7 in these locations. Finally, stereotaxic injection of individual cytokines into the hindbrain or spinal cord revealed region‐ and cytokine‐specific modulation of localizing cue expression by astrocytes. These findings identify a role for inflammatory cytokines in mediating local astrocyte‐dependent mechanisms of immune cell trafficking within the CNS during neuroinflammation.

## Graphical abstract

- Regional astrocyte heterogeneity mediates autoimmune neuroinflammation.
- Th17‐associated cytokines regulate VCAM‐1 on brain stem astrocytes.
- IFNγ upregulates CXCR7 on spinal cord astrocytes to facilitate T cell entry.

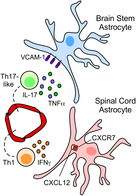

## 1 INTRODUCTION

In the inflammatory and neurodegenerative disease multiple sclerosis (MS), and one of its animal models, experimental autoimmune encephalomyelitis (EAE), T cells become activated and incite an autoimmune attack on the myelin sheath, which leads to inflammatory lesions causing motor and sensory function loss (Frohman, Racke, & Raine, 2006). Symptoms of MS vary widely between individuals and can include physical disability, largely thought to arise from spinal cord involvement (Losseff et al., 1996), urinary tract deficits, implicating lesions in the pons (Weissbart et al., 2017), and depression, which is correlated to temporal lobe lesions (Siegert & Abernethy, 2005). While specific subsets of symptoms are linked to distinct central nervous system (CNS) regions, how immune cells are targeted to those regions remains unclear.

Using EAE as a model for MS, cytokine expression, and T cell phenotype have been strongly linked to lesion location (Lees, Golumbek, Sim, Dorsey, & Russell, 2008; Pierson & Goverman, 2017; Simmons, Liggitt, & Goverman, 2014; Stromnes, Cerretti, Liggitt, Harris, & Goverman, 2008). While myelin‐specific, IFNγ‐expressing Th1 cells target the spinal cord in classical EAE, IFNγ‐deficient T cells, and Th17 cells preferentially traffic to the hindbrain, causing atypical EAE (Lees et al., 2008; Stromnes et al., 2008). Briefly, during classical EAE mice exhibit ascending disease including loss of tail tonicity, ataxia, and hind limb paralysis associated with mononuclear cell infiltration of spinal cord white matter tracts. Loss of IFNγ signaling results in atypical EAE wherein perivascular accumulation and parenchymal infiltration of mononuclear immune cells occurs primarily the brain stem. Mice with atypical EAE exhibit dystonic limb dysfunction and disturbances in coordination/equilibrium (Domingues, Mues, Lassmann, Wekerle, & Krishnamoorthy, 2010; Kroenke, Chensue, & Segal, 2010; Lees et al., 2008; Stromnes et al., 2008). Several other cytokines also have critical roles in MS and EAE pathogenesis including IL‐1β (Lin et al., 2016; Rossi et al., 2014) and TNFα (Gimenez, Sim, & Russell, 2004; Rossi et al., 2014), affecting the encephalitogenic capacity of T cells and their ability to induce parenchymal inflammation. These cytokines also have a role in upregulating chemokines and their receptors, in addition to vascular adhesion molecules at the blood–brain barrier (BBB) that together facilitate immune cell entry into the CNS (Cayrol et al., 2008; Cruz‐Orengo et al., 2011; Shrikant, Chung, Ballestas, & Benveniste, 1994; Simmons et al., 2014; Williams, Holman, & Klein, 2014). Previously, we demonstrated that the inflammatory cytokines IL‐1β, IL‐17, and IFNγ have differential effects on the bioavailability of CXCL12, a CNS localizing chemokine, via regulation of its scavenger receptor, CXCR7 (Cruz‐Orengo, Holman, et al., 2011) and that TNFR1 signaling is required for astrocytic expression of vascular cell adhesion molecule (VCAM)‐1 (Gimenez et al., 2004), which facilitates parenchymal CNS leukocyte infiltration (Hurwitz, Lyman, Guida, Calderon, & Berman, 1992; Rosenman, Shrikant, Dubb, Benveniste, & Ransohoff, 1995; Shrikant et al., 1994; Winkler & Beveniste, 1998). While specific T cell localizing cues that target distinct T cell subtypes to CNS regions have been described (Glatigny, Duhen, Arbelaez, Kumari, & Bettelli, 2015; Glatigny, Duhen, Oukka, & Bettelli, 2011; Rothhammer et al., 2011), roles for astrocytes in guiding T cell localization to specific locations during autoimmunity have not been defined.

Astrocytes are known to exhibit regional heterogeneity (Zhang & Barres, 2010), particularly in response to cytokines during neuroinflammation (Daniels et al., 2017; Simmons et al., 2014), and are reactive within MS lesions (Correale & Farez, 2015; Ponath et al., 2017; Ponath, Park, & Pitt, 2018). Since astrocytes within the neurovascular unit are positioned to interact with and respond to CNS‐infiltrating immune cells, we wondered if regional responsiveness to cytokines might differentially influence the expression of T cell localizing cues and thereby contribute to region‐specific inflammation. Here, we demonstrate that Th1 and Th17 cytokines impact CNS region‐specific leukocyte localizing cues during autoimmune neuroinflammation via differential responses of astrocytes. in vitro studies using murine and human astrocytes revealed that upregulation of CNS localizing cues is both cytokine‐ and region‐specific. Modeling region‐specific T cell trafficking in vivo, we found that expression of VCAM‐1 and CXCR7 is differentially regulated by astrocytes exposed to Th1 versus Th17 cytokines. Further, stereotactic injection of cytokine into the hindbrain or spinal cord of naïve animals induced region‐ and cytokine‐specific expression of localizing cues. These findings identify a role for inflammatory cytokines in modulating local astrocyte‐dependent mechanisms of immune cell entry during neuroinflammation.

## 2 MATERIALS AND METHODS

### 2.1 EAE induction

Eight‐ to nine‐week‐old C57Bl/6 WT, *Ifngr1* ^−/−^ and *Ifng* ^*−/−*^ mice were obtained from The Jackson Laboratory and *Cxcr7* ^GFP/+^ mice were bred and maintained in‐house. *Cxcr7* ^GFP/+^ *Ifngr1* ^−/−^ *and Vcam1* ^fl/fl^ *Gfap*‐Cre^+^ mice were generated in the laboratory of Robyn S. Klein and *Ifngr1* ^fl/fl^ *Gfap*‐Cre^+^ were generated in the laboratory of Jessica L. Williams. All mice used in experiments were maintained in specific pathogen‐free conditions. WT, *Ifng* ^−/−^ or Thy1.1^+^ MOG_35‐55_‐specific Th1 clones were generated as previously described (Lees et al., 2008) and 10^7^ cells were transferred to naïve recipients via retroorbital injection. Active immunization and clinical scoring were performed as previously described (Cruz‐Orengo et al., 2011). All animal studies were performed in accordance with the Animal Care and Use Committee guidelines of the National Institutes of Health and were conducted under protocols approved by the Animal Care and Use Committee of Washington University and the Cleveland Clinic.

### 2.2 CNS leukocyte isolation and flow cytometric analysis

Following cardiac perfusion with PBS, cells were isolated from the spinal cords of WT or *Ifngr1* ^−/−^ mice. Briefly, tissue was digested in HBSS (Gibco) containing 0.05% collagenase D (Sigma), 0.1 μg/ml TLCK trypsin inhibitor (Sigma), 10 μg/ml DNaseI (Sigma), and 10 mM HEPES pH 7.4 (Gibco) for 1 hr at room temperature. Tissue was pushed through 70 μM strainer to create a single cell suspension and then separated via 37% Percoll (GE Healthcare) in order to remove myelin debris. Cells cultured in vitro were washed with PBS following stimulation. Cells were stained with fluorescently conjugated antibodies to CD4, Thy1.1, and CD49d. Prior to intracellular staining, all cells were stimulated with PMA and ionomycin in the presence of brefeldin A for 3 hr, permeabilized and labeled with fluorescently conjugated antibodies to IL‐1β, IL‐17, GM‐CSF, TNFα, and IFNγ as previously described (McCandless, Wang, Woerner, Harper, & Klein, 2006). Data were collected using an LSR II flow cytometer (BD) and analyzed using the FlowJo software (Tree Star).

### 2.3 Real‐time quantitative (q) RT‐PCR on human and murine astrocytes

Primary adult human brain stem and spinal cord astrocytes were obtained from ScienCell and grown according to provided protocols in complete ScienCell Astrocyte Medium. Briefly, primary human astrocytes were isolated from normal brain stem or spinal cord tissue and at P0 were tested for morphology by phase contrast and relief contrast microscopy and GFAP positivity by immunofluorescence. Cell number, viability (≥70%), and proliferative potential (≥15 pd) were also assessed and negative screening for potential biological contaminants was confirmed prior to cryopreservation and receipt of frozen cells at P1. All experiments were performed using astrocytes at P1 or P2. Murine astrocytes were isolated from mixed glia cultures from the brain stem or spinal cord of postnatal Day 3–5 WT pups as previously described (Patel, McCandless, Dorsey, & Klein, 2010). Once confluent, cells were treated with indicated cytokines (10 ng/ml) for 24 hr. Following exposure to indicated cytokines (10 ng/ml) for 24 hr, total RNA was collected using the RNeasy kit (QIAGEN) according to the manufacturer's instructions. qRT‐PCR was performed using *Power* SYBR® Green PCR master mix (ThermoFisher) and primers specific for human *CXCR7* (forward: GGC TAT GAC ACG CAC TGC TAC A, reverse: TGG TTG TGC TGC ACG AGA CT), *VCAM1* (forward: GAT ACA ACC GTC TTG GTC AGC CC, reverse: CAG TTG AAG GAT GCG GGA GTA TAT G), *IL1R* (forward: GAG ATG GAG ACT TCC TGC C, reverse: GTC ACA TCA CAG GAC ACG G), *IL17R* (forward: CTA AAC TGC ACG GTC AAG AAT, reverse: CTG AGC TCA TGC ATG GCG TGG), *TNFR1* (forward: TTC TGT ACC AAG ACC TCG, reverse: CAG ATC TGT AAC GTG GTG), and *IFNGR1* (forward: CAG AAT GGA TTG ATG CCT GC, reverse: GGC ATA CAG CAA ATT CTT CTT). For murine astrocytes, primers specific for *Cxcr7* (forward: GGT CAG TCT CGT GCA GCA TA, reverse: GTG CCG GTG AAG TAG GTG AT) and *Vcam1* (forward: CAA ATC CTT GAT ACT GCT CAT, reverse: TTG ACT TCT TGC TCA CAG C) were used. Calculated copies were normalized to human *GAPDH* or murine *Gapdh* copy number as previously described (Klein et al., 2005). All primers are listed 5′‐3′.

### 2.4 Western blot analysis

Human brain stem and spinal cord astrocytes (ScienCell) were seeded in six‐well plates until confluent and treated with media alone or recombinant human cytokine for 24 hr. Protein lysate (20 μg) was isolated using RIPA buffer supplemented with a protease and phosphatase‐3 inhibitor cocktail (Sigma‐Aldrich). Lysates were resolved on a 4–12% Tris gel and transferred onto a PVDF transfer membrane (Invitrogen) using an iBlot2 system according to standard protocols. Blots were probed with polyclonal rabbit anti‐VCAM‐1 or ‐CXCR7 (ThermoFisher) and monoclonal mouse anti‐β‐actin (ThermoFisher) antibodies, followed by incubation with appropriate HRP‐conjugated secondary antibodies (ThermoFisher). Blots were imaged using a BioRad ChemiDoc MP imaging system.

### 2.5 Immunocytochemistry on murine astrocytes

Astrocytes were isolated from mixed glia cultures from the brain stem or spinal cord of postnatal Day 3–5 WT or *Cxcr7* ^GFP/+^ pups as previously described (Patel et al., 2010). Once confluent, cells were treated with indicated cytokines (10 ng/ml) for 24 hr and labeled as previously described (Williams, Patel, Daniels, & Klein, 2014). Briefly, cells were fixed with 4% PFA, labeled with anti‐GFP (1:1,000, Invitrogen) or VCAM‐1 (1:200, Thermo Scientific) antibodies. Secondary antibodies conjugated to Alexa 488 (1:500, Molecular Probes) were applied for 1 hr at room temperature and nuclei were counterstained with DAPI (Molecular Probes). Cells were imaged using the 60x oil objective of a Zeiss LSM 880 confocal laser scanning microscope. Thresholds were set using appropriate isotype controls and mean positive area was analyzed using the AxioVision (Carl Zeiss) image analysis software.

### 2.6 Stereotaxic injection

Stereotaxic injections were performed by Washington University's animal surgery core using a David Koft stereotaxic frame. Adult male and female Cxcr7^GFP/+^ animals were shaved and anesthetized with 2% isoflurane prior to surgery. The surgical site was cleaned with 75% ethanol, followed by 1% betadine. A 1.0 cm sagittal incision was made at the level of L2/L3 vertebrae for spinal cord injections or at the brain stem. A glass needle was inserted at the incision site using the Drummond Nanoject2. Stereotaxic injections were performed at a depth of 0.5 mm, just lateral to midline. A volume of 0.25 μl recombinant mouse TNFα (25 ng, R&D Systems), recombinant mouse IFNγ (25 ng, Biolegend), or vehicle (PBS) was injected. After microinjection, the dorsal muscle was sutured with 4–0 silk. The incision skin was pulled together using forceps and sutured using 4.0 nylon. The mice were kept on a heating pad until awake. Animals were sacrificed 72 hr postinjection.

### 2.7 IHC on murine CNS tissue

Frozen sections were prepared and detection of cell markers were accomplished as previously described (Williams, Patel, et al., 2014). Briefly, tissue sections were blocked with goat serum and triton x (Sigma) for 1 hr at room temperature and then exposed to anti‐CD3 (1:100, Biolegend), ‐CD31 (1:100, R&D Systems), ‐GFAP (1:200, Invitrogen), ‐GFP (1:1,000, Invitrogen), or ‐CXCL12β (1:20, eBioscience) overnight at 4°C. Primary anti‐VCAM‐1 (1:500, Thermo Scientific) was applied after an additional block using the mouse on mouse detection kit (Vector Laboratories) according to manufacturer's instructions. Secondary antibodies conjugated to Alexa 488 or Alexa 555 (1:400, Molecular Probes) were applied for 1 hr at room temperature. Nuclei were counterstained with DAPI (Molecular Probes). Sections were analyzed using the 40× water objective of a Zeiss LSM 880 confocal laser scanning microscope. Thresholds were set using appropriate control antibodies and the mean Mander's coefficient was determined using the ImageJ software.

### 2.8 In vivo CCX771 treatment

About 30 mg/kg CCX771 (ChemoCentryx) or vehicle (10% captisol) was administered s.c. to mice daily following the onset of EAE in 100 μl of vehicle throughout the treatment period as previously described (Williams, Patel, et al., 2014).

### 2.9 Statistical analysis

Data were analyzed using the Prism software (GraphPad). Data were analyzed with parametric tests (two‐tailed Student's *t* test or one‐ or two‐way ANOVA), with correction for multiple comparisons where appropriate. Clinical EAE data were analyzed by Mann–Whitney *U* test. A *p* value of less than .05 was considered statistically significant. Data are expressed as means ± SEM. Sample sizes are indicated in the figure legends.

## 3 RESULTS

### 3.1 Cytokines dictate T cell regionality in the CNS

**Figure 1:**
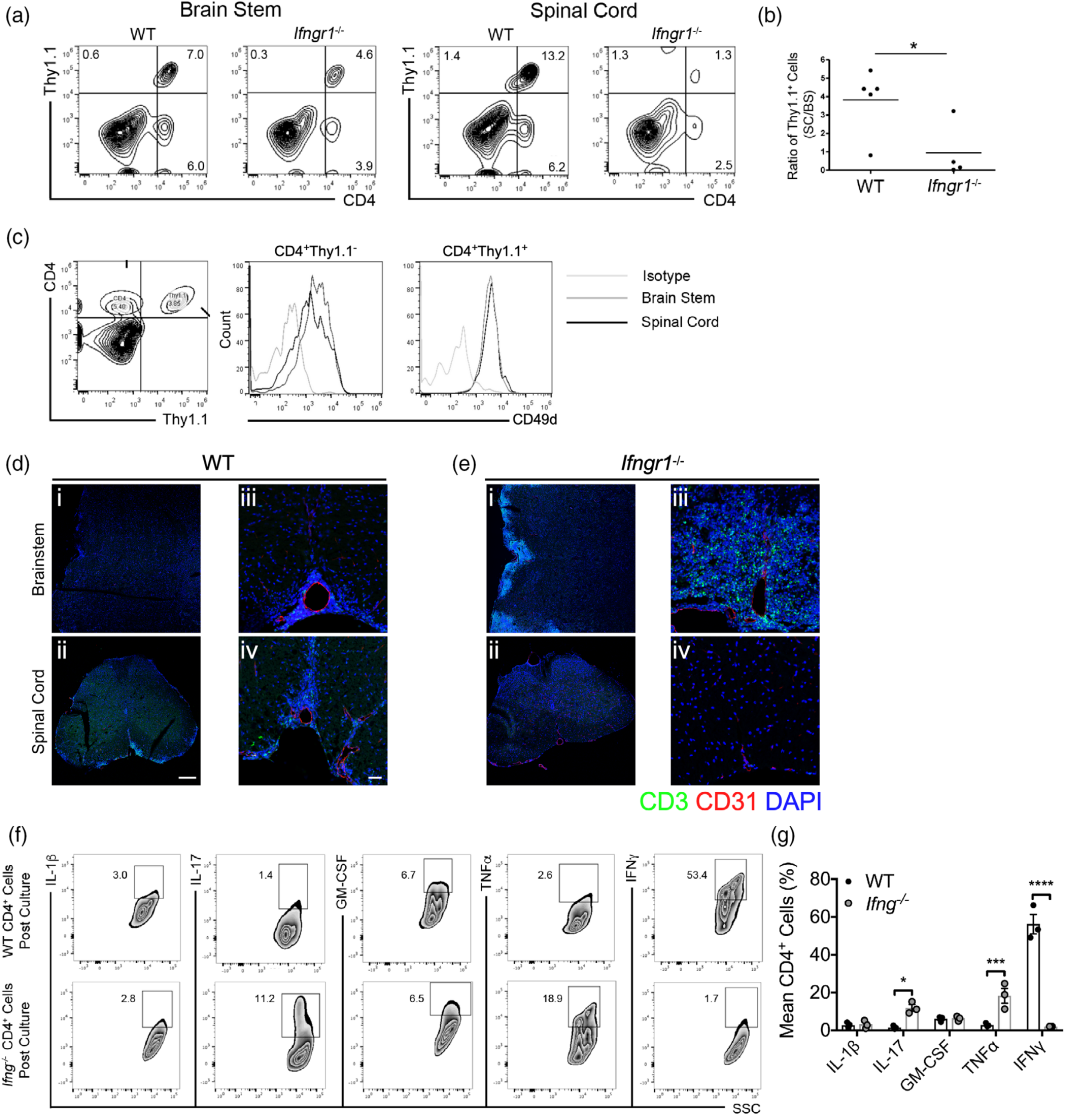
Cytokines dictate regional T cell trafficking within the CNS. (a) Activated, myelin‐specific Thy1.1^+^ T cell clones were injected into WT or *Ifngr1* ^−/−^ recipients. CNS leukocytes were collected during peak disease (10 days post‐transfer) and the presence of donor and recipient T cells was analyzed. (b) The number of spinal cord Thy1.1^+^ cells was calculated and normalized to the number of Thy1.1^+^ cells isolated from the brain stem. (c) The level of CD49d on donor and recipient CD4^+^ T cells isolated from the brain stem (gray line) and spinal cord (black line) was analyzed and compared to an isotype control (light gray line) by flow cytometry. (d) Activated, myelin‐specific WT T cells were injected into naïve WT and (e) *Ifngr1* ^−/−^ recipients and at peak EAE. CNS tissue was cryopreserved then labeled for the expression of CD3 and CD31. A representative, (i and ii) tiled 20× magnification image; scale bar = 200 μm, and (iii and iv) a 20× image are shown; scale bar = 50 μm. (f) Leukocytes were isolated from the draining lymph nodes of immunized WT or *Ifng* ^−/−^ mice and restimulated in the presence of MOG_35‐55_ for 72 hr. Following restimulation, intracellular cytokine expression in CD4^+^ cells was analyzed by flow cytometry. (g) Cytokine expression in CD4^+^ cells was quantified over three independent experiments. *p < .05; ***p < .001, ****p < .0001 by Student's *t* test or two‐way ANOVA. CNS, central nervous system; EAE, experimental autoimmune encephalomyelitis

Given the known role of IFNγ in targeting T cells to the spinal cord versus the hindbrain during classical versus atypical EAE induced by adoptive transfer (Lees et al., 2008), we determined whether IFNGR1 signaling in recipient animals contributes to differential T cell trafficking. Transfer of myelin‐specific, Thy1.1^+^ Th1 cells into WT or *Ifngr1* ^−/−^ hosts revealed that IFNγ signaling is required for the accumulation of donor T cells in the spinal cord, but not the brain stem (Figure 1a). Selective IFNGR1‐mediated retention of T cells in the spinal cord over the brain stem was confirmed via comparison of numbers of lymphocytes isolated from each region at peak disease (Figure 1b). Previous studies indicate that differential T cell expression of α4 integrin (CD49d), which binds VCAM‐1, is responsible for spinal cord entry of Th1 cells (Glatigny et al., 2011; Rothhammer et al., 2011); however, we found no difference in CD49d expression by donor or recipient CD4^+^ cells isolated from the spinal cord versus the brain stem during EAE (Figure 1c). To determine the anatomical location of adoptively transferred WT T cells, we performed IHC analysis on recipient tissue. In the brain stem of WT recipients, infiltrating cells were primarily cuffed around the brain stem vasculature; however, in the spinal cord, infiltrating cells are observed within the parenchyma (Figure 1d). Further, while few infiltrating cells were found in spinal cords of *Ifngr1* ^−/−^ mice, a vast number of cells were observed around the vasculature and infiltrating the parenchyma (Figure 1e). Next, we characterized cytokine expression patterns of restimulated myelin‐specific WT T cells that traffic to the spinal cord versus *Ifng* ^−/−^ T cells, which preferentially target the hindbrain. WT, Th1‐driven cells primarily express IFNγ, and *Ifng* ^−/−^ cells expressed more IL‐17 and TNFα (Figure 1f,g). These data suggest that T cell cytokine profile is associated with localization to specific CNS region.

### 3.2 Regional astrocyte localization cues are regulated by cytokines

**Figure 2:**
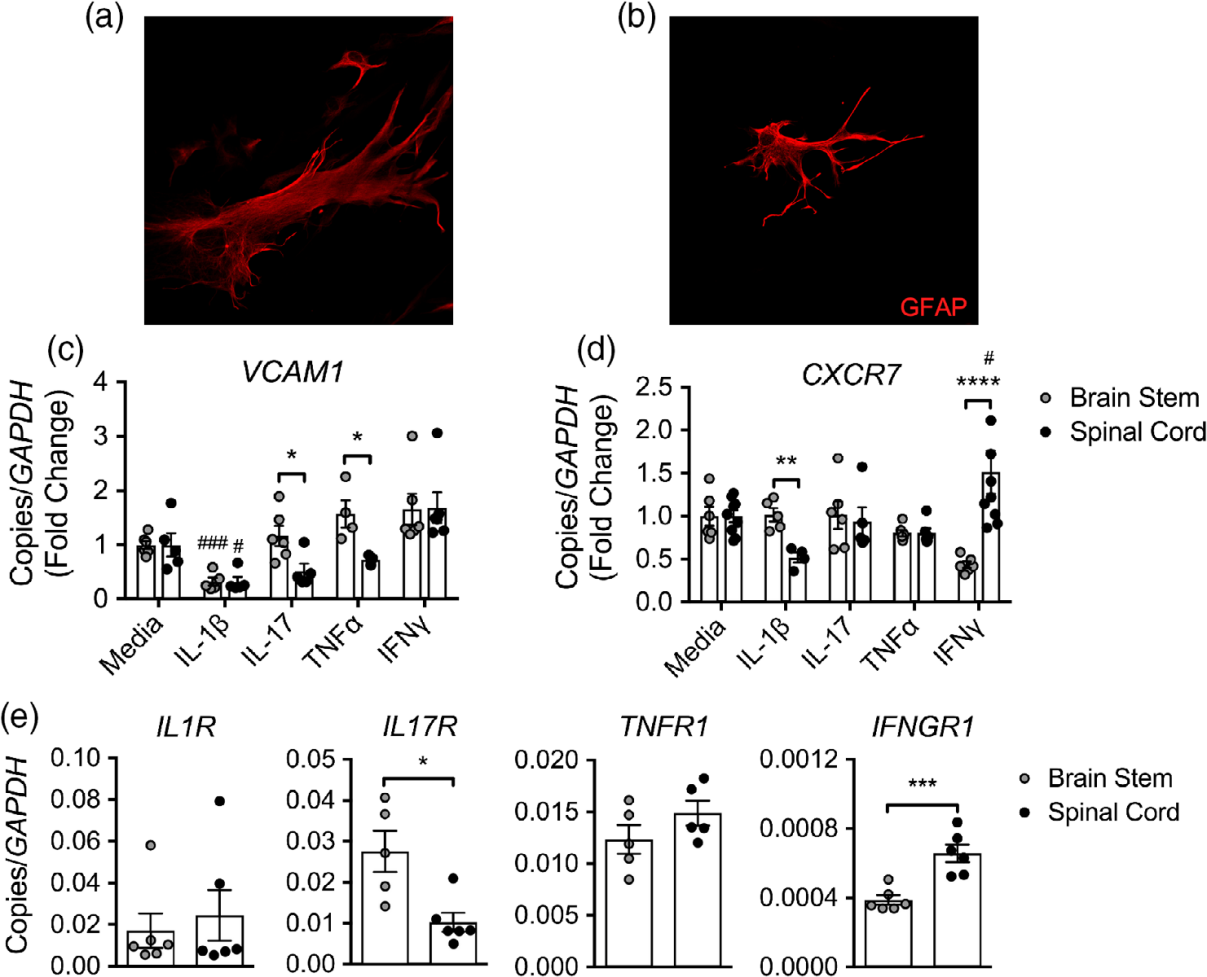
Cytokines mediate regional localization cues on astrocytes. Primary human astrocytes from the (a) brain stem and (b) spinal cord were labeled for GFAP by ICC and imaged using confocal microscopy at 20× magnification. (c) *CXCR7* and (d) *VCAM1* mRNA levels in primary adult human brain stem and spinal cord astrocytes following exposure to 10 ng/ml of recombinant cytokine for 24 hr. (e) Cytokine receptor expression in primary adult human brain stem and spinal cord astrocytes was quantified by qRT‐PCR following culture in astrocyte medium. Data shown are combined results from two independent experiments with *n* = 5–8 per treatment. *p < .05; **p < .01; ***p < .001, ****p < .0001 between region and ^#^ *p* < .05; ^###^ *p* < .001 compared to media‐treated astrocytes by Student's *t* test or two‐way ANOVA. ICC, immunocytochemistry

Astrocytes form a complex network surrounding the CNS endothelium, help to maintain barrier properties, respond highly to cytokines, and may express a variety of molecules involved in T cell localization, including CXCL12, CXCR7, and VCAM‐1 (Abbott, Patabendige, Dolman, Yusof, & Begley, 2010; Gimenez et al., 2004; Patel et al., 2012; Rosenman et al., 1995; Williams, Patel, et al., 2014). We previously demonstrated that CXCL12 scavenging by CXCR7 at the BBB regulates spinal cord infiltration and EAE disease severity (Cruz‐Orengo, Chen, et al., 2011; Cruz‐Orengo, Holman, et al., 2011), and that astrocyte expression of CXCR7 similarly regulates extracellular levels of CXCL12 (Williams, Patel, et al., 2014). To examine the effect of cytokines on expression of regional astrocytic T cell localizing cues, we exposed primary adult human astrocytes (Figure 2a,b) to T cell cytokines that target inflammation to the brain stem or spinal cord, followed by detection of *VCAM1* and *CXCR7* mRNA transcript (Figure 1f,g). Treatment with IL‐1β reduced *VCAM1* transcripts in astrocytes derived from either CNS region, but decreased *CXCR7* mRNA levels only in spinal cord astrocytes (Figure 2c,d). Further, IL‐17 and TNFα increased *VCAM1* transcript in brain stem compared to spinal cord astrocytes (Figure 2c), while IFNγ highly upregulated *CXCR7* in spinal cord astrocytes compared to those from the brain stem (Figure 2d). While granulocyte‐macrophage colony‐stimulating factor (GM‐CSF) is also known to contribute to inflammation during EAE and MS (Galli et al., 2019; Kroenke et al., 2010; Pierson & Goverman, 2017; Rothhammer et al., 2011), we did not observe a significant regional difference in *VCAM1* transcripts in astrocytes following GM‐CSF treatment (data not shown).

To determine whether differential responses to cytokines were due to region‐specific baseline expression levels of cytokine receptors, we performed quantitative (q)RT‐PCR analyses of receptor levels in primary human astrocytes. Consistent with work by Simmons et al. that suggests brain astrocytes are dependent on IL‐17 signaling for specific chemokine induction (Simmons et al., 2014), q‐PCR analysis of transcript levels revealed increased expression of *IL17R* in untreated human brain stem astrocytes, while astrocytes from the spinal cord had increased *IFNGR1* expression at baseline (Figure 2e). Together, these data suggest that Th1 cytokines influence the localizing chemokine axis CXCL12/CXCR7 on spinal cord astrocytes, while Th17‐associated cytokines preferentially upregulate VCAM‐1 on brain stem astrocytes, and that these differential responses may be due to enhanced cytokine receptor expression in astrocytes within these CNS regions.

### 3.3 Th17‐associated cytokines mediate VCAM‐1 expression on brain stem astrocytes

**Figure 3:**
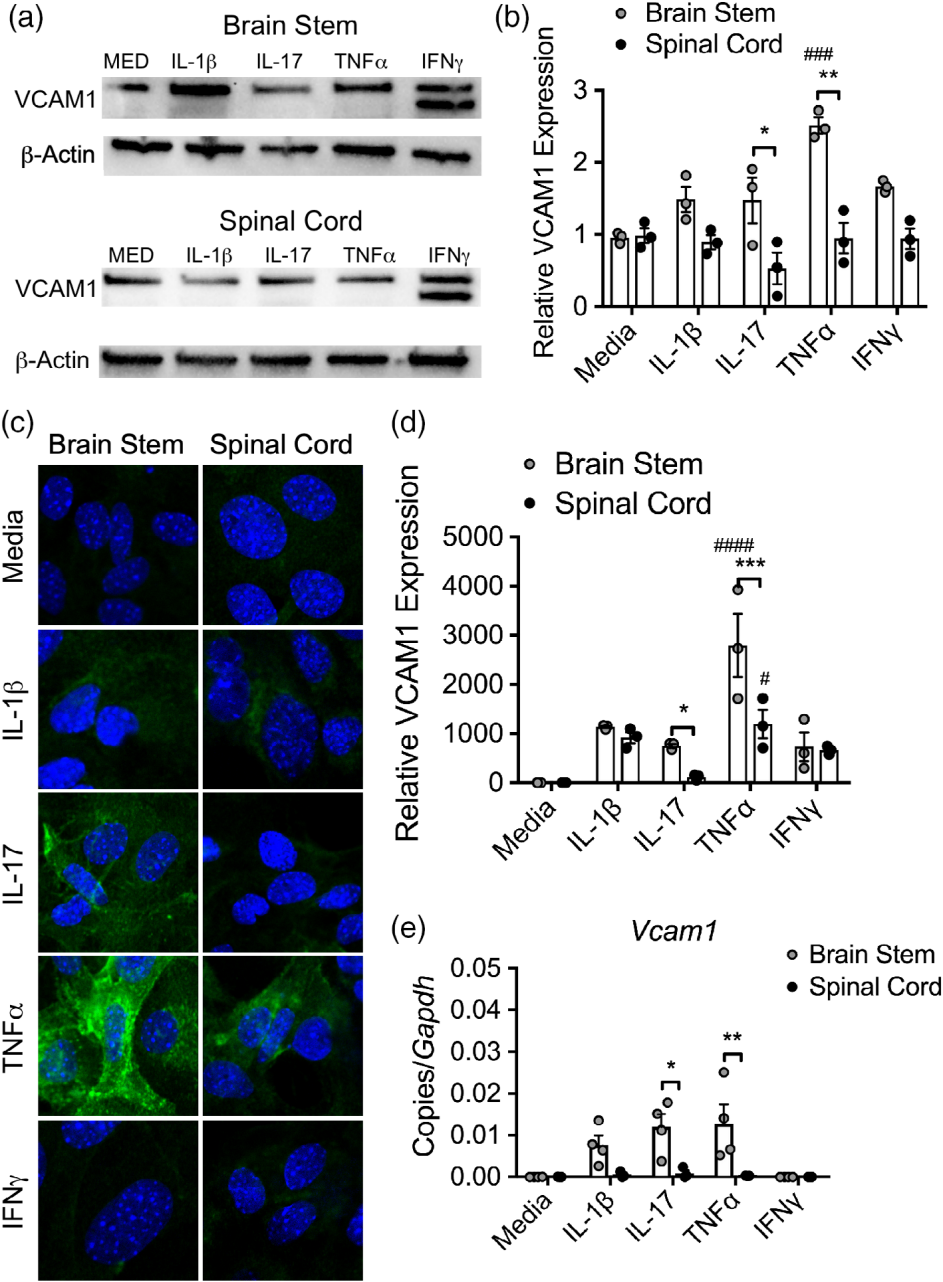
Brain stem astrocytes preferentially upregulate VCAM‐1 in response to Th17‐associated cytokines. (a) WB analysis of lysate from human brain stem and spinal cord astrocytes following exposure to 10 ng/ml cytokine for 24 hr was probed for VCAM‐1. (b) VCAM‐1 density was quantified and normalized to media controls. (c) Primary murine astrocytes from the brain stem and spinal cord of neonatal pups were exposed to 10 ng/ml of recombinant cytokine for 24 hr, fixed and analyzed for VCAM‐1 expression by confocal microscopy. (d) The level of expression was quantified using the AxioVision image analysis software and normalized to media‐treated astrocytes. (e) Following cytokine exposure, mRNA transcript was isolated from murine astrocytes and *Vcam1* expression was measured by qRT‐PCR. Data shown are combined results from three to four independent experiments. *p < .05; **p < .01; ***p < .001 between region and ^#^ *p* < .05; ^###^ *p* < .001 compared to media‐treated astrocytes by two‐way ANOVA. WB, western blot

To investigate Th17 cytokine‐mediated, regional expression of VCAM‐1, we exposed brain stem and spinal cord astrocytes to IL‐1β, IL‐17, TNFα, and IFNγ and assessed VCAM‐1 protein levels in human astrocytes by western blot (WB; Figure 3a,b) and in murine astrocytes by immunocytochemistry (Figure 3c,d) and qRT‐PCR (Figure 3e). Using all assays, VCAM‐1 protein or transcript expression levels were consistently enhanced in brain stem astrocytes treated with IL‐17 or TNFα compared to similarly treated astrocytes derived from the spinal cord (Figure 3). Of note, a ~70 kDa isoform of VCAM‐1 thought to mediate localization of polarized cells to specific surface domains was upregulated in human astrocytes following IFNγ exposure (Montes‐Sanchez et al., 2009; Pirozzi, Terry, & Labow, 1994; Rosenman et al., 1995; Figure 3a).

**Figure 4:**
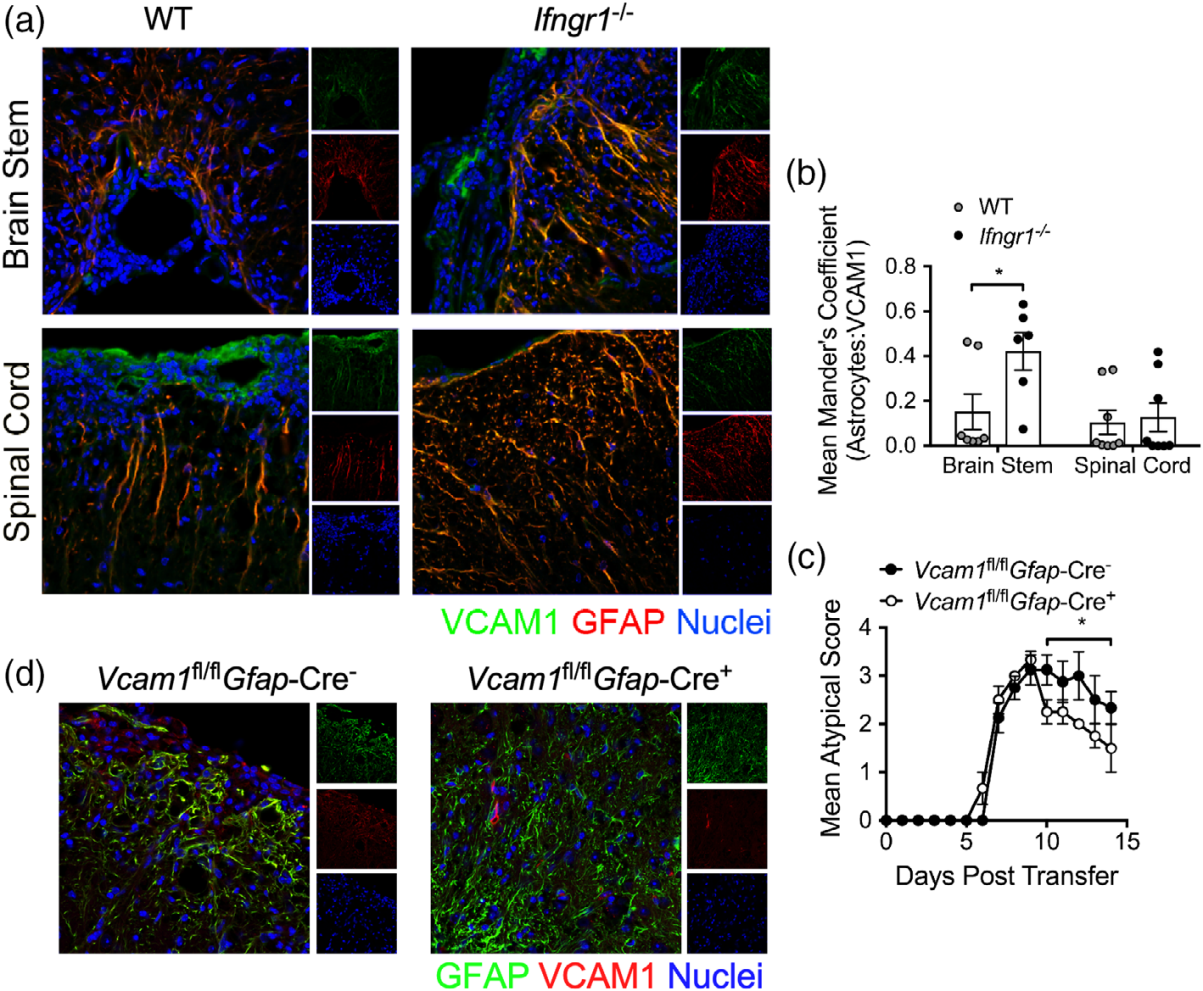
Brain stem inflammation is maintained by VCAM‐1 on astrocytes during EAE. (a) Activated WT, myelin‐specific Th1 clones were injected into WT or *Ifngr1* ^−/−^ mice and the CNS was prepared for IHC at peak disease. The brain stem and spinal cord of recipient mice were labeled for VCAM‐1 and GFAP expression. (b) The mean Mander's coefficient for colocalization (ImageJ) was used to quantify astrocyte‐associated VCAM‐1 expression in each CNS region. Data are representative of two independent experiments and data points represent individual animals. (c) Activated, myelin‐specific *Ifng* ^−/−^ T cells were injected into *Vcam1* ^fl/fl^ *Gfap*‐Cre^−^ or *Vcam1* ^fl/fl^ *Gfap*‐Cre^+^ littermates and monitored for signs of atypical EAE. (d) Representative 20× confocal images of brain stem tissue from *Vcam1* ^fl/fl^ *Gfap*‐Cre^−^ and *Vcam1* ^fl/fl^ *Gfap*‐Cre^+^ littermates labeled for IHC to confirm reduced VCAM‐1 expression in GFAP^+^ astrocytes. *p < .05 by two‐way ANOVA or Mann–Whitney *U* test for EAE clinical scores. CNS, central nervous system; EAE, experimental autoimmune encephalomyelitis; IHC, immunohistochemistry

To examine if astrocyte VCAM‐1 is upregulated in vivo during region‐specific neuroinflammation, we induced classical and atypical EAE by transferring activated, myelin‐specific WT T cells into WT or *Ifngr1* ^−/−^ hosts, respectively. Analysis of CNS lesions at peak disease revealed that VCAM‐1 expression in astrocytes was significantly elevated in the brain stem of mice with atypical EAE compared to those with classical EAE (Figure 4a,b). To confirm the role of VCAM‐1 expression on brain stem astrocytes, we induced atypical EAE by injecting activated, *Ifng* ^−/−^ T cells into *Vcam1* ^fl/fl^ *Gfap*‐Cre^+^ and *Vcam1* ^fl/fl^ *Gfap*‐Cre^−^ littermates. While Cre^+^ and Cre^−^ mice displayed similar induction of EAE, Cre^+^ mice exhibited significantly improved recovery (Figure 4c) in the absence of VCAM‐1 expression in GFAP^+^ astrocytes (Figure 4d). Taken together, these data suggest that VCAM‐1 upregulation on brain stem astrocytes by Th17‐associated cytokines has a key role in preventing recovery from CNS autoimmunity.

### 3.4 Interferon‐γ signaling induces CXCR7 expression specifically in spinal cord astrocytes

**Figure 5:**
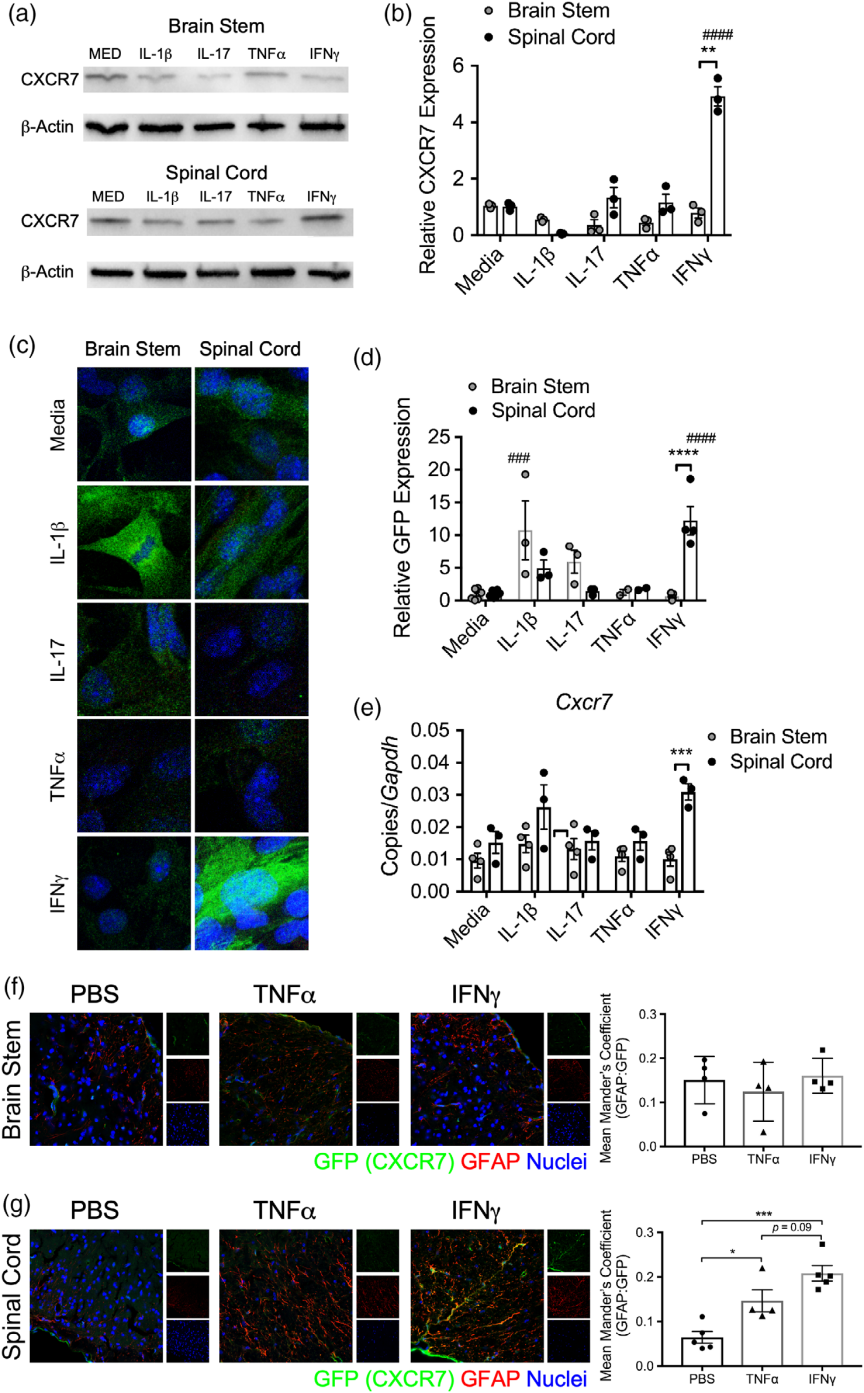
Spinal cord astrocytes express CXCR7 in response to IFNγ. (a) WB analysis of lysate from human brain stem and spinal cord astrocytes following exposure to 10 ng/ml cytokine for 24 hr was probed for CXCR7. (b) CXCR7 density was quantified and normalized to media controls. (c) Primary murine astrocytes from the brain stem and spinal cord of neonatal *Cxcr7* ^GFP/+^ pups were exposed to 10 ng/ml of recombinant cytokine for 24 hr, fixed and analyzed for GFP expression by confocal microscopy. (d) The level of GFP expression was quantified using the AxioVision image analysis software and normalized to media‐treated astrocytes. (e) Following cytokine exposure, mRNA transcript was isolated from murine astrocytes and *Cxcr7* expression was measured by qRT‐PCR. Data shown are combined results from three to four independent experiments. About 25 ng of recombinant mouse TNFα, IFNγ, or vehicle was injected into the brain stem or spinal cord of *Cxcr7* ^GFP/+^ mice. About 72 hr following injection, brain stem and spinal cord sections were harvested and prepared for IHC. (e) Brain stem and (f) spinal cord sections were stained and analyzed for expression of GFP and GFAP. The mean Mander's coefficient for colocalization (ImageJ) was used to quantify astrocyte‐associated GFP. Data are pooled from two independent experiments per CNS region. *p < .05; ***p < .001 by two‐way ANOVA. CNS, central nervous system; IHC, immunohistochemistry; WB, western blot

CXCR7 acts as a scavenger receptor to remove extracellular CXCL12, a localizing cue for T cells at the BBB (Cruz‐Orengo, Holman, et al., 2011; Williams, Patel, et al., 2014). To determine if astrocytes exhibit cytokine‐ and region‐specific alterations in CXCR7 expression, we exposed human brain stem and spinal cord astrocytes to IL‐1β, IL‐17, TNFα, and IFNγ and assessed CXCR7 protein levels. WB analysis revealed that CXCR7 levels were induced by IFNγ specifically in astrocytes derived from the spinal cord (Figure 5a,b). Using regional astrocytes isolated from *Cxcr7* ^GFP/+^ reporter mice, we confirmed that IFNγ was the only cytokine tested that elevated CXCR7 levels in spinal cord, but not brain stem astrocytes (Figure 5c,d). Although IL‐1β treatment increased CXCR7 above that of media‐treated, there was not a regional difference detected (Figure 5d). Analysis of transcript expression levels of *Cxcr7* in murine astrocytes treated with cytokine confirmed these findings (Figure 5e).

To test whether IFNγ promoted CXCR7 expression in astrocytes in vivo, we stereotaxically injected either PBS, TNFα, or IFNγ into the brain stem or spinal cord of *Cxcr7* ^GFP/+^ mice and measured GFP colocalization within GFAP‐expressing astrocytes. Importantly, while astrocyte CXCR7 expression was unchanged in the brain stem after cytokine injections, (Figure 5f), TNFα and IFNγ significantly increased astrocyte CXCR7 expression above that of the PBS‐treated group in the spinal cord (Figure 5g). These data suggest that although TNFα and IFNγ may modulate adhesion molecule expression on astrocytes, the localizing cues downstream of specific cytokine signaling is region‐specific.

### 3.5 Interferon‐γ‐mediated CXCR7 expression is critical for spinal cord, but not brain stem autoimmune neuroinflammation

**Figure 6:**
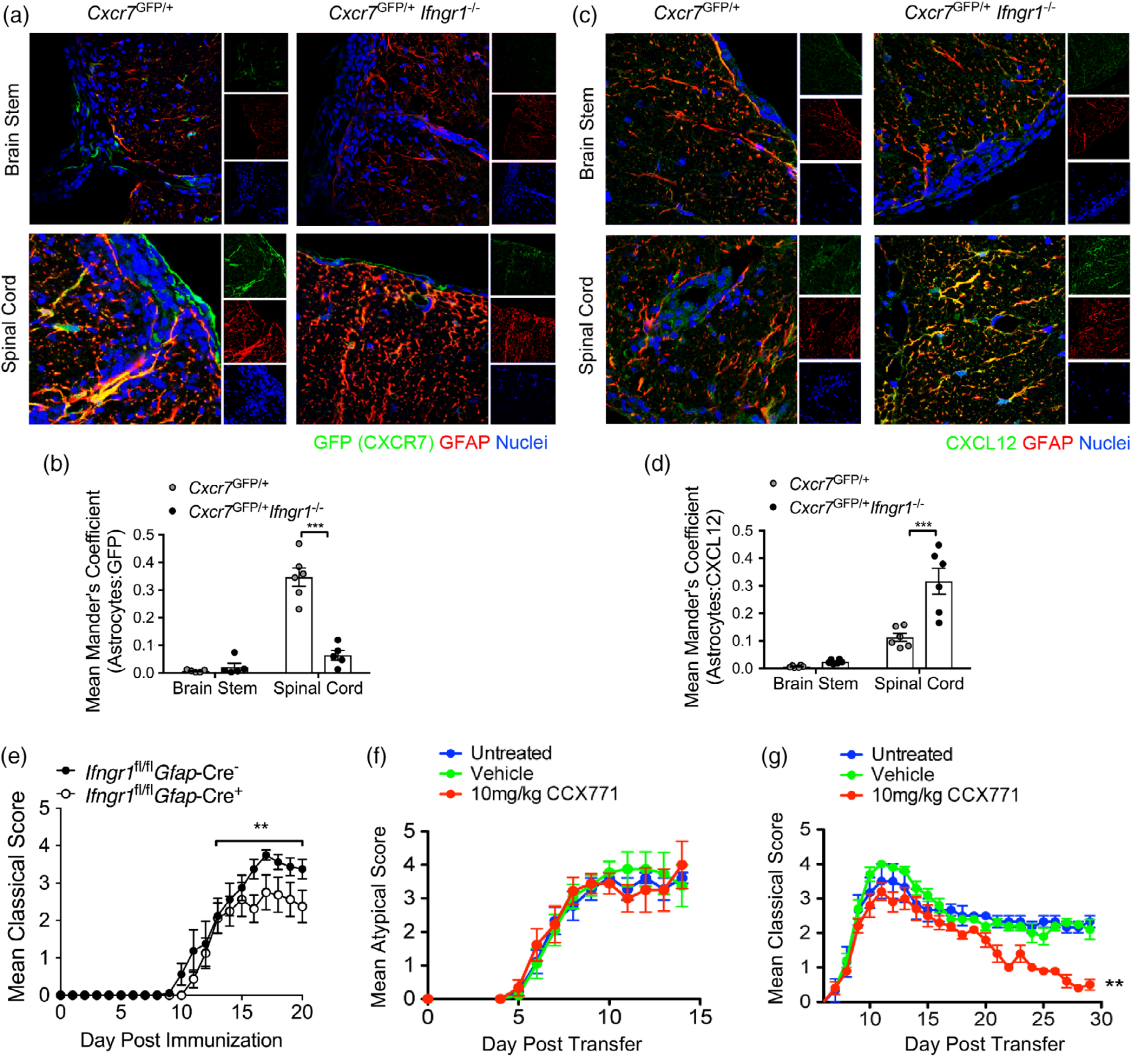
Astrocyte IFNγ signaling regulates the CXCR7/CXCL12 chemokine axis to maintain spinal cord inflammation. (a–d) Activated WT, myelin‐specific Th1 clones were injected into either *Cxcr7* ^GFP/+^ or *Cxcr7* ^GFP/+^ *Ifngr1* ^−/−^ mice and the CNS was prepared for IHC at peak disease. The brain stem and spinal cord of recipient mice were labeled for GFAP and (a) GFP or (c) CXCL12. The mean Mander's coefficient for colocalization (ImageJ) was used to quantify astrocyte‐associated (b) GFP and (d) CXCL12 expression in each CNS region. Data are representative of two independent experiments. (e) *Ifngr1* ^fl/fl^ *Gfap*‐Cre^−^ or *Ifngr1* ^fl/fl^ *Gfap*‐Cre^+^ littermates were immunized with MOG_35‐55_ and adjuvants and monitored for signs of classical EAE. Data are representative of three independent experiments with *n* = 5–8 mice per group. Activated (f) WT or (g) *Ifng* ^−/−^ myelin‐specific T cells were injected into WT mice. At the onset of EAE, mice were either untreated or subcutaneously injected with 100 μl captisol or 10 mg/kg CCX771 in 100 μl captisol daily. (f) Classical or (g) atypical signs of EAE were monitored. Data are representative of two to three independent experiments with *n* = 4–7 mice per group. ***p < .001, by two‐way ANOVA and **p < .01 by Mann–Whitney *U* test for EAE clinical scores. CNS, central nervous system; EAE, experimental autoimmune encephalomyelitis; IHC, immunohistochemistry

To assess if region‐specific CXCR7 is upregulated in vivo during autoimmune neuroinflammation, we induced atypical or classical EAE by transferring myelin‐specific WT T cells to *Cxcr7* ^GFP/+^ or *Cxcr7* ^GFP/+^ *Ifngr1* ^−/−^ hosts, respectively. Detection of CXCR7 during EAE using these reporter mice revealed increased, astrocyte‐specific expression of CXCR7 in the spinal cord of mice with intact IFNGR1 signaling compared to *Cxcr7* ^GFP/+^ *Ifngr1* ^−/−^ recipients (Figure 6a,b). To ensure this increase in spinal cord CXCR7 was biologically relevant, we also analyzed CXCL12 levels and found a corresponding decrease (Figure 6b–d), consistent with a loss of T cell tethering by astrocytes and enhanced parenchymal infiltration and damage (Cruz‐Orengo, Holman, et al., 2011). Further, a reduction in disease severity in mice with astrocyte‐specific deletion of *Ifngr1* following induction of classical EAE (Figure 6e) confirmed that IFNγ signaling in astrocytes is critical for the perpetuation of spinal cord inflammation during EAE. Next, since CXCR7 induction by IFNγ was spinal cord specific, we wondered if CXCR7 antagonism during atypical, hindbrain‐affected EAE would prove to be ineffective, as opposed to its beneficial function in classical EAE (Cruz‐Orengo, Chen, et al., 2011; Cruz‐Orengo, Holman, et al., 2011). To test this, we transferred either myelin‐specific *Ifng* ^−/−^ or WT cells to induce atypical or classical EAE, respectively. Following the onset of EAE, mice were treated daily with CCX771, a small molecule antagonist of CXCR7 as previously described (Cruz‐Orengo, Chen, et al., 2011; Cruz‐Orengo, Holman, et al., 2011; Williams, Patel, et al., 2014). As expected, CCX771 had efficacy only in mice with spinal cord inflammation (Figure 6g) and had no effect on EAE course in hindbrain‐affected animals (Figure 6f). These data suggest that astrocyte IFNγ signaling has a critical role in modulating the CXCR7/CXCL12 chemokine axis to promote T cell entry specifically into the spinal cord during EAE.

## 4 DISCUSSION

Although the roles of chemokines, integrins, and vascular adhesion molecules on T cell subtypes during EAE pathogenesis have been described, the contribution of CNS cells, particularly astrocytes, in regional T cell trafficking has not been extensively investigated. Here, we show that T cell cytokine profile is critical for the upregulation of region‐specific localizing cues on astrocytes. While Th17‐associated cytokines preferentially upregulated VCAM‐1 on brain stem astrocytes, the prototypical Th1 cytokine, IFNγ, modulated the CXCL12/CXCR7 axis for T cell entry into the spinal cord. In the absence of IFNγR signaling, brain stem astrocytes upregulated VCAM‐1 expression and astrocyte‐specific deletion of VCAM‐1 reduced the severity of atypical EAE, suggesting IFNγ may partially limit T cell entry into the brain stem during EAE by inhibiting the expression of VCAM‐1 on astrocytes. Loss of IFNγR signaling also resulted in dampened CXCR7 expression in the spinal cord, leading to an increase in CXCL12, which according to our previous findings, likely mediated retention of T cells in the perivascular space, contributing to reduced EAE severity. Using a small molecule inhibitor of CXCR7, we confirmed this was a spinal cord‐specific phenomenon as CXCR7 inhibition failed to ameliorate atypical, brain stem‐associated EAE, but was highly effective in classical, spinal cord‐involved EAE, as previously shown (Cruz‐Orengo, Holman, et al., 2011).

Cytokines are key mediators of EAE pathogenesis and are known to regulate adhesion molecule and localizing cue expression on a number of CNS cells, including astrocytes. While transfer of WT and TNFR1‐deficient T cells results in similar EAE severity and T cell trafficking was independent of TNFR1 signaling on T cells, loss of TNFα responsiveness in the CNS results in a lack of VCAM‐1 expression on astrocytes and an inability of T cells to migrate into the spinal cord (Gimenez et al., 2004). Further, transfer of WT Th1 cells into IFNGR1‐deficient mice results in T cell invasion of the hindbrain, but not the spinal cord, which is identical to the site of neuroinflammation induced after the transfer of *Ifng* ^−/−^ T cells into WT hosts (Lees et al., 2008). Thus, disruption of cytokine signaling significantly impacts T cell trafficking during EAE pathogenesis in a region‐specific fashion.

The role of IFNγ in MS and EAE has been a paradox for more than three decades. Many early studies describe a solely proinflammatory and pathologic function in disease (Fletcher, Lalor, Sweeney, Tubridy, & Mills, 2010; Olsson, 1992); however, more recent evidence supports additional protective roles, suggesting IFNγ has complex, stage‐dependent pleiotropic effects in MS and EAE (Arellano, Ottum, Reyes, Burgos, & Naves, 2015; Bever Jr., Panitch, Levy, McFarlin, & Johnson, 1991; Furlan et al., 2001; Kelchtermans, Billiau, & Matthys, 2008; Miller, Wang, Tan, & Dittel, 2015; Ottum, Arellano, Reyes, Iruretagoyena, & Naves, 2015; Sosa, Murphey, Robinson, & Forsthuber, 2015; Sun et al., 2017). Classically, IFNγ is thought to drive acute autoimmune neuroinflammation via promotion of Th1 responses and regulation of peripheral immune cell infiltration into the CNS (Kuchroo et al., 2002; Lee, Chanamara, Pleasure, & Soulika, 2012; Legroux & Arbour, 2015; Nylander & Hafler, 2012; Zhu, Yamane, & Paul, 2010). Indeed, early clinical trials using intravenous IFNγ likely failed because participants had clinically definite RRMS, which is inflammatory in nature (Panitch, Hirsch, Haley, & Johnson, 1987). Additionally, astrocytes treated with IFNγ can promote the proliferation and differentiation of myelin‐specific Th1 cells (Dong & Benveniste, 2001) and upregulate adhesion molecules to facilitate CNS immune cell entry during early disease (Rosenman et al., 1995; Tan, Gordon, Mueller, Matis, & Miller, 1998). However, during chronic disease, there is accumulating evidence that IFNγ has a protective role during EAE and MS. In progressive MS patients, improving symptoms correlated with high levels of serum IFNγ, while patients with clinical worsening had relatively low levels of serum IFNγ (Bever Jr. et al., 1991). In EAE, systemic or intraventricular administration of IFNγ in mice and marmosets during chronic phases reduced disease severity, demyelination, and mortality (Billiau et al., 1988; Jagessar et al., 2012; Voorthuis et al., 1990), and significantly delayed relapses in a murine model of chronic‐relapsing EAE (Heremans, Dillen, Groenen, Martens, & Billiau, 1996). Likewise, neutralizing IFNγ exacerbated disease and made an EAE‐resistant mouse strain susceptible (Billiau et al., 1988; Duong, Finkelman, Singh, & Strejan, 1994; Duong, St Louis, Gilbert, Finkelman, & Strejan, 1992; Heremans et al., 1996; Lublin et al., 1993; Voorthuis et al., 1990). Further, only mice with intact IFNγ signaling were able to recover from EAE (Willenborg, Fordham, Staykova, Ramshaw, & Cowden, 1999). Using a signaling deficient dominant‐negative *Ifngr1* driven by the *Gfap* promotor, the protective effects of IFNγ signaling during chronic EAE were linked to astrocytes. These mice exhibited exacerbated clinical disease, inflammation, and demyelination only during the chronic phase of EAE, suggesting IFNγ signaling in astrocytes may provide stage‐specific protection (Hindinger et al., 2012). Our study is complimentary to these and other findings in that we describe a pathogenic role for IFNγ signaling in astrocytes during acute EAE. Future studies will further dissect the protective nature of IFNγ signaling in astrocytes during chronic EAE.

In addition to exerting proinflammatory effects, many localizing molecules are constitutively expressed and regulate migration of peripheral immune cells during immune surveillance as well as during neuroinflammation (Williams, Holman, & Klein, 2014). For example, CXCR7 is constitutively expressed throughout the CNS and on the endothelium of the BBB (Cruz‐Orengo et al., 2014; McCandless et al., 2006) along with its ligand, CXCL12 (Li & Ransohoff, 2008), suggesting a role for this chemokine/receptor pair in immune cell trafficking into perivascular spaces during homeostatic conditions. During neuroinflammation, such as in EAE and MS, however, scavenging of CXCL12 from the BBB surface as well as disrupted polarity of CXCL12 across CNS endothelium promotes immune cell infiltration into the CNS parenchyma (Cruz‐Orengo et al., 2014; Cruz‐Orengo, Holman, et al., 2011; McCandless et al., 2008). Adhesion molecules are also implicated in parenchymal T cell infiltration during CNS autoimmunity including α4 integrin, which is required for Th1 cells to enter the spinal cord (Glatigny et al., 2011; Rothhammer et al., 2011), and αLβ2 integrin, which is required for the CNS entry of *Ifng* ^−/−^ Th17 cells (Rothhammer et al., 2011). Additionally, Th17 cells utilize the CCR6‐CCL20 axis to enter the lumbar spinal via the choroid plexus (Reboldi et al., 2009). Like Th17 cells, T regulatory cells also rely on αL integrin for CNS homing during autoimmunity (Glatigny et al., 2015). The differential CNS homing requirements of T cell subsets in conjunction with region‐specific responses of astrocytes to their cytokines may underlie the relative ratios of pro‐ versus anti‐inflammatory effects during CNS autoimmunity.

Astrocytes exert both pathologic and protective roles during neuroinflammation and neurodegeneration. During acute demyelination in MS, progressive multifocal leukoencephalopathy, metachromatic leukodystrophy, and subacute infarction, astrocytes remove myelin via receptor‐mediated endocytosis, which results in activation of the transcription factor NF‐κB and further release of chemokines (Ponath et al., 2017). An astrocytic risk variant associated with increased NF‐κB activation has also been identified, and drives lymphocyte recruitment with increasing lesion size, contributing to local autoimmune inflammation (Brambilla et al., 2009; Ponath et al., 2018). In Alzheimer's disease, plaque‐associated astrocytes may become activated and eliminate Aβ, but it is unclear if this is a protective mechanism or contributes to pathogenesis and secondary plaque formation (Garwood et al., 2017; Nagele et al., 2004). Astrocytes also critically impact recovery in several models of neurodegeneration. Fragmented mitochondria released by damaged neurons may be removed by neighboring astrocytes (Davis et al., 2014). Alternatively, in a model of stroke, astrocytes were observed to transfer functional mitochondria to neurons, contributing to their recovery (Hayakawa et al., 2016). In vitro, astrocyte CCL6 protects neurons from excitotoxic cell death via a phosphatidylinositol‐3 kinase‐dependent pathway (Nakagawa, Izumi, Takada‐Takatori, Akaike, & Kume, 2019). Finally, there is emerging evidence to suggest that altered astrocyte function may contribute to Parkinson's disease, but the underlying mechanisms have yet to be demonstrated (Booth, Hirst, & Wade‐Martins, 2017). Taken together, it is clear that astrocytes play pivotal roles in neurologic diseases at multiple stages of pathogenesis and repair. In summary, our findings contribute to our understanding of astrocyte roles in CNS autoimmunity by demonstrating that inflammatory T cell cytokines modulate local astrocyte‐guided entry of T cells, providing new insights for the development of CNS region‐targeted therapies for patients with specific patterns of neuroinflammation.

## CONFLICT OF INTEREST

The authors declare no competing financial interests.

## AUTHOR CONTRIBUTIONS

Conceptualization: J.L.W., J.H.R., and R.S.K.; methodology: J.L.W., S.M., B.C.S., J.H.R., and R.S.K.; analysis: J.L.W., S.M., B.C.S., and J.S.; investigation: J.L.W., J.S., B.P.D., and S.M.; writing of the original draft: J.L.W.; review and editing of the manuscript: J.L.W., S.M., B.P.D., and R.S.K.; supervision and funding acquisition: J.L.W., J.H.R., and R.S.K.

## Data Availability

The data that support the findings of this study are available from the corresponding author upon reasonable request.
